# Functions of Muscarinic Receptor Subtypes in Gastrointestinal Smooth Muscle: A Review of Studies with Receptor-Knockout Mice

**DOI:** 10.3390/ijms22020926

**Published:** 2021-01-18

**Authors:** Yasuyuki Tanahashi, Seiichi Komori, Hayato Matsuyama, Takio Kitazawa, Toshihiro Unno

**Affiliations:** 1 Department of Advanced Life Sciences, Faculty of Life Sciences, Kyoto Sangyo University, Kyoto 603-8555, Japan; ytanavet@cc.kyoto-su.ac.jp; 2 Laboratory of Veterinary Pharmacology, Department of Veterinary Medicine, Faculty of Applied Biological Sciences, Gifu University, 1-1 Yanagido, Gifu 501-1193, Japan; gkyakuri@yahoo.co.jp (S.K.); mhayato@gifu-u.ac.jp (H.M.); 3 Department of Veterinary Science, Rakuno Gakuen University, Ebetsu, Hokkaido 069-8501, Japan; tko-kita@rakuno.ac.jp

**Keywords:** muscarinic receptor subtypes, knockout mouse, smooth muscle, gastrointestinal tract, non-selective cationic channels, signal transduction pathways

## Abstract

Parasympathetic signalling via muscarinic acetylcholine receptors (mAChRs) regulates gastrointestinal smooth muscle function. In most instances, the mAChR population in smooth muscle consists mainly of M_2_ and M_3_ subtypes in a roughly 80% to 20% mixture. Stimulation of these mAChRs triggers a complex array of biochemical and electrical events in the cell via associated G proteins, leading to smooth muscle contraction and facilitating gastrointestinal motility. Major signalling events induced by mAChRs include adenylyl cyclase inhibition, phosphoinositide hydrolysis, intracellular Ca^2+^ mobilisation, myofilament Ca^2+^ sensitisation, generation of non-selective cationic and chloride currents, K^+^ current modulation, inhibition or potentiation of voltage-dependent Ca^2+^ currents and membrane depolarisation. A lack of ligands with a high degree of receptor subtype selectivity and the frequent contribution of multiple receptor subtypes to responses in the same cell type have hampered studies on the signal transduction mechanisms and functions of individual mAChR subtypes. Therefore, novel strategies such as genetic manipulation are required to elucidate both the contributions of specific AChR subtypes to smooth muscle function and the underlying molecular mechanisms. In this article, we review recent studies on muscarinic function in gastrointestinal smooth muscle using mAChR subtype-knockout mice.

## 1. Introduction

Muscarinic acetylcholine receptors (mAChRs) are widely expressed at presynaptic and postsynaptic sites throughout the body, where they regulate many critical neurological and physiological processes, including neural excitability, cardiac and smooth muscle contraction and endocrine and exocrine gland activity. The mAChR family consists of five molecularly distinct subtypes, M_1_–M_5_, all of which are coupled to membrane-associated GTP-binding proteins (G-proteins) that transduce binding of acetylcholine (ACh) and other muscarinic agonists into various intracellular signalling cascades [1,2,3,4]. Molecular biological studies have shown that the M_2_ and M_4_ subtypes are preferentially coupled to G-proteins of the pertussis toxin (PTX)-sensitive G_i/o_ family, whereas M_1_, M_3_ and M_5_ are selectively coupled to the PTX-insensitive G_q/11_ family [3]. However, more precise identification of specific functions in individual cell types and tissues has been challenging due to the paucity of muscarinic receptor subtype-specific antibodies for immunohistochemistry [5], the lack of commercially available and highly subtype-selective ligands, the complex overlapping expression patterns of individual subtypes and the contributions of multiple subtypes to the same cellular or physiological response. To circumvent these problems, many mAChR subtype-specific knockout (KO) mouse strains have been generated by gene targeting technology [6,7]. As disruption of one specific mAChR gene appears to have little substantial effect on the expression levels of the remaining four mAChRs [8], these models are providing crucial evidence for the contributions of specific mAChR subtypes to various developmental, physiological and pathophysiological processes.

In the gastrointestinal tract and many other visceral organs, release of ACh from autonomic nerves triggers excitation and contraction of smooth muscle by activating mAChRs. Smooth muscle mAChRs are a mixture of M_2_ and M_3_ subtypes with M_2_ predominance (M_2_:M_3_ = 3–5:1) [1,9], although mRNAs encoding all five mAChR subtypes have been detected in gastrointestinal smooth muscle [10]. Activation of mAChRs triggers multiple biochemical and electrical signalling events that modulate contraction [11,12,13,14]. Traditional studies using various mAChR antagonists suggested that the M_3_ subtype is the primary mediator of contraction in visceral smooth muscles, while the contribution of the M_2_ subtype was considered less clear [15,16]. However, more recent studies have demonstrated that the M_2_ subtype modulates contraction, at least in part by inhibiting cyclic AMP (cAMP)-dependent relaxation [9] and by regulating smooth muscle ion channel activity [17,18,19]. Nevertheless, little is known as to which mAChR subtype mediates the individual cellular events that underlie or modulate the contractile response.

In this article, we review key results from studies using mAChR subtype-specific KO mice to examine contributions to the excitation and contraction of smooth muscle and the underlying molecular signalling pathways. In addition to the contributions of post-junctional mAChRs expressed by smooth muscle cells, pre-junctional mAChRs expressed by various enteric neurons contribute to regulation of smooth muscle activity through modulation of excitatory or inhibitory neurotransmitters, including ACh, substance P and nitric oxide (NO). The roles of neural mAChR subtypes have been studied using mAChR-mutant mice [20,21] and reviewed [22].

## 2. Adenylyl Cyclase Inhibition and Phosphoinositide Hydrolysis

A common feature of mAChR activation in gastrointestinal smooth muscle is the co-induction of two second messenger responses, adenylyl cyclase inhibition resulting in reduced accumulation of cAMP and stimulation of phospholipase C (PLC) hydrolysis of phosphoinositides resulting in formation of inositol 1,4,5-trisphosphate (InsP_3_) and diacylglycerol (DAG). In turn, IP_3_ releases Ca^2+^ from intracellular stores and DAG activates protein kinase C (PKC), leading to the phosphorylation of various proteins [1,9,15]. Further, muscarinic subtypes demonstrate complex reciprocal modulation of second messenger pathways.

Sakamoto et al. (2007) [23] examined effects of the potent non-subtype-selective muscarinic agonist carbachol on accumulation of cAMP elicited by the β-adrenoceptor agonist isoprenaline, which activates adenylyl cyclases, in ileal longitudinal muscles from mAChR-KO and wild-type mice. Carbachol (1 µM) induced greater inhibition of isoprenaline-stimulated cAMP accumulation in ileal muscle from M_3_-KO mice than wild-type mice (50% vs. 26%). In M_2_-KO mice, however, carbachol enhanced isoprenaline-stimulated cAMP accumulation by ~30%, while M_2_/M_3_ double-KO mice exhibited only a slight increase. These results are consistent with a pharmacological study in rabbit stomach suggesting that M_2_ receptors are coupled to inhibition of adenylyl cyclases via G_αi3_ and that M_3_ receptors are coupled to adenylyl cyclase activation via G_βγq/11_ [24]. Thus, the cAMP response to carbachol in wild-type mice reflects the predominant inhibitory influence of M_2_ receptors.

Tran et al. (2006) [25] investigated the coupling of mAChR subtypes to phosphoinositide hydrolysis in ileum and urinary bladder smooth muscles from mAChR-KO mice by measuring agonist-mediated conversions of [^3^H]inositol-labelled phosphoinositides into [^3^H]inositol phosphates in response to the potent muscarinic agonist oxotremorine-M. Phosphoinositide hydrolysis in the urinary bladder did not differ markedly between M_2_-KO and wild-type mice during oxotremorine-M stimulation, and no measurable response was observed in bladder tissue from M_3_-KO or M_2_/M_3_ double-KO mice. These results are consistent with a previous study of guinea-pig urinary bladder suggesting that the muscarinic phosphoinositide response is exclusively M_3_-mediated [26]. On the other hand, in the ileum, M_1_, M_2_ and M_3_ subtypes appeared to participate in the oxotremorine-M-induced phophoinositide response [25]. In this tissue, M_2_-KO mice exhibited small and M_3_-KO mice displayed large decreases in phosphoinositide hydrolysis, while M_2_/M_3_ double-KO mice still demonstrated a substantial response, similar to an M_1_ profile in competitive antagonism experiments. The relative contributions of M_1_, M_2_ and M_3_ subtypes to the wild-type phosphoinositide response amounted to 15%, 5% and 80%, respectively, suggesting a major role for the M_3_ receptor and minor roles for the M_1_ and M_2_ receptors in mouse ileum, which is generally consistent with multiple pharmacological studies suggesting that M_3_ mediates phosphoinositide hydrolysis via coupling to G_q/11_ proteins [9,27]. Alternatively, M_2_ likely stimulates PLC due to βγ dimer released from G_i_ proteins [28]. The M_1_-mediated response likely occurs in ileal smooth muscle cells rather than associated cells such as enteric neurons since oxotremorine-M also induced robust phosphoinositide hydrolysis in dissociated muscle cells from M_2_/M_3_ double-KO ileum [25]. However, the resultant second messengers InsP_3_ and DAG appear unlikely to be involved in contraction and other muscarinic effects, including intracellular Ca^2+^ release, membrane depolarisation, activation or inhibition of various ion channels and Ca^2+^ sensitisation of contractile proteins, because all were totally abolished by knockout of M_2_, M_3_ or both, as described below. Further study using M_1_-KO mice will be needed to confirm the contribution of M_1_-mediated phosphoinositide hydrolysis to gastrointestinal smooth muscle contraction.

## 3. Muscarinic Regulation of Ion Channel Activity

Multiple effects of mAChR activation on ion channels have been described in gastrointestinal and other visceral smooth muscles, including activation of non-selective cationic channels or chloride (Cl^−^) channels, inhibition or potentiation of voltage-dependent Ca^2+^ channels (VDCCs) and modulation of several K^+^ channel types [12,14,29,30]. These effects underlie muscarinic modulation of smooth muscle excitation and contraction, as evidenced by recent studies of ileal longitudinal smooth muscle cells from various mAChR-KO mice.

### 3.1. Activation of Non-Selective Cationic Channels

**Figure 1:**
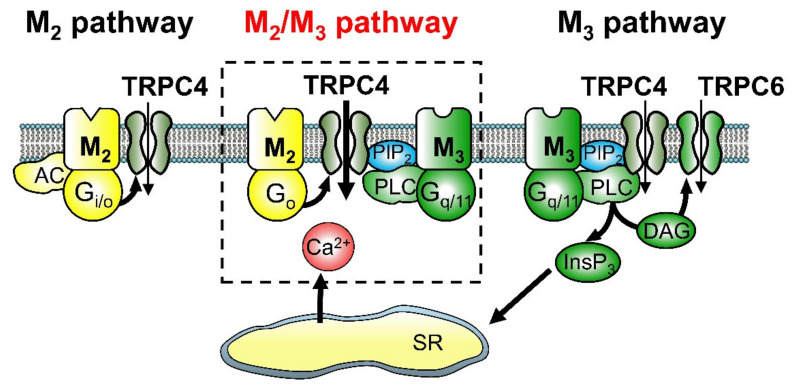
Three distinct muscarinic signalling pathways leading to transient receptor potential (TRP)-like cationic channel opening in ileal myocytes (redrawn from Tanahashi et al. 2020 [39]). The M_3_/G_q/11_/phospholipase C (PLC) pathway activates brief opening states of 70-pS and 120-pS cationic channels and concurrently evokes InsP_3_-induced Ca^2+^ release. Opening of the lower conductance channel is induced by relief from PIP_2_ inhibition following PLC-mediated hydrolysis, while the higher conductance channel is activated by PLC-generated diacylglycerol (DAG). The M_2_ pathway transmits M_2_ signals via G_i/o_ proteins to the 70-pS channel, which shifts the gating state from the brief to a longer opening mode, and also inhibits adenylyl cyclase. The M_2_/M_3_ pathway transmits M_2_ signals via G_o_ protein, and M_3_ signals via G_q/11_/PLC, to the 70-pS cationic channel, resulting in channel gating with a much longer open mode. This pathway is the major contributor to the generation of *mIcat*, but is inactive when either the M_2_ or M_3_ receptor is absent, or when either G_o_, G_q/11_ or PLC is inactivated. In other words, the activity of this pathway is conditional, occurring only when both M_2_/G_o_ and M_3_/G_q/11_ signalling pathways are activated. Studies of mAChR-KO mice [23,44] and TRPC-mutant mice [42] indicate that these 70-pS and 120-pS cationic channel activities are mediated by TRPC4 and TRPC6, respectively. The M_2_/M_3_ pathway, but not the M_2_ or M_3_ pathway, involves a signalling step in which Ca^2+^ has a potentiating effect on TRPC channel activation, suggesting that the M_3_ pathway may facilitate M_2_/M_3_ pathway function through InsP_3_-induced Ca^2+^ release. Whether the M_2_/M_3_ pathway has a significant role in stimulating InsP_3_/DAG formation or inhibiting cAMP accumulation is currently unclear. One study suggested that G_o_ protein is not involved in adenylyl cyclase inhibition by M_2_ receptors of intestinal smooth muscle [24]. These three pathways may also converge on voltage-dependent Ca^2+^ channels (VDCCs) to suppress Ca^2+^ influx via the same G-protein pathways mediating cationic channel activation (see Figure 10B in Tanahashi et al., 2009 [49]).

The opening of non-selective cationic channels is the primary mechanism by which mAChR activation produces smooth muscle depolarisation and contraction [11,31]. Early studies of guinea-pig ileal myocytes reported that the mAChR-mediated cationic current (*mIcat*) was sensitive to PTX and potentiated strongly by a rise in intracellular Ca^2+^ concentration ([Ca^2+^]_i_) [32,33,34], suggesting activation primarily by M_2_ receptors and potentiation by M_3_-mediated Ca^2+^ release. However, accumulating evidence now suggests that channel opening is a mixed M_2_/M_3_ response requiring co-activation of both receptors. Under conditions where [Ca^2+^]_i_ was buffered to eliminate the influence of Ca^2+^ release, *mIcat* was inhibited by M_2_-preferring and M_3_-preferring antagonists, but also markedly depressed by anti-G_αo_ antibody and the PLC inhibitor U-73122 [17,35,36,37]. Furthermore, *mIcat* was blocked by YM-254890, a chromobacterium-derived peptide that specifically inhibits G_q/11_ protein signalling activity [38] (see Figures 1 and 3 in Tanahashi et al., 2020 [39]). Sakamoto et al. (2006) [40] reported that *mIcat* in mouse ileal myocytes showed all of the typical pharmacological features of guinea pig *mIcat*. Therefore, it has been hypothesised that the *mIcat* of both species is activated through an interaction between M_2_ and M_3_ receptors and ensuing activation of downstream signalling pathways via coupling to G_o_ and G_q/11_ proteins, respectively.

To evaluate this hypothesis, Sakamoto et al. (2007) [23] measured carbachol-evoked *mIcat* in ileal myocytes from M_2_-KO, M_3_-KO, M_2_/M_3_ double-KO and wild-type mice under conditions where Ca^2+^ modulation was eliminated. In M_2_-KO and M_3_-KO myocytes, *mIcat* amplitudes evoked by a maximally effective carbachol dose (100 µM) were only 11% and 6% of wild-type amplitude, respectively, and current was undetectable in M_2_/M_3_ double-KO myocytes. However, all mutants demonstrated normal G-protein-cationic channel coupling as judged from their ability to generate cationic currents in response to infusion of the direct G-protein activator GTPγS. Taken together, these results provide direct evidence that the wild-type *mIcat* is not a simple additive response from M_2_ and M_3_ activation but results from a synergy between M_2_- and M_3_-mediated signalling pathways.

Single channel activity was also measured in outside-out membrane patches excised from M_2_-KO, M_3_-KO and wild-type ileal myocytes stimulated with carbachol [23]. Activated patches from wild-type myocytes showed three distinct patterns of cationic channel activity. One was characterised by 70-pS unitary conductance channels with three distinct open states (as distinguished by mean open times (Oτ values) = 0.25, 1.1 and 13.2 ms) and mimicked by active patches from M_3_-KO cells, indicating induction by M_2_. The second pattern consisted of mixed bursts of brief 70-pS and 120-pS unitary currents, each with single open states (Oτ = 0.46 and 0.32 ms, respectively) and mimicked by activated patches from M_2_-KO cells, indicating induction by M_3_. Finally, the third pattern was characterised by longer 70-pS unitary currents with four distinct open states (Oτ = 0.62, 2.7, 16.9 and 121.1 ms) undetected in active patches from M_2_-KO or M_3_-KO myocytes. Therefore, this channel activity requires M_2_/M_3_ interaction. These single channel analyses provide evidence that intact ileal myocytes are endowed with three distinct muscarinic cationic channel activation pathways initiated by activation of M_2_ or M_3_ and co-activation of M_2_ and M_3_, with the major contribution of the M_2_/M_3_ pathway to *mIcat* activity in intact ileal myocytes. Thus, deletion of M_2_ or M_3_ subtypes resulted in abolition of not only each separate pathway but also the M_2_/M_3_ synergistic pathway.

Transient receptor potential canonical (TRPC) channels are widely assumed to mediate G-protein-coupled non-selective cationic currents in smooth muscle [41]. Recently, Tsvilovskyy et al. (2009) [42] provided compelling evidence that TRPC4 and TRPC6 channels are responsible for *mIcat* in ileal myocytes, with relative contributions of 84% and ~19%, respectively, at a holding potential of −50 mV based on electrophysiological studies of TRPC4-KO, TRPC6-KO and TRPC4/6 double-KO mice. A similar result was obtained in gastric myocytes [43]. Single channel recordings by Tsvilovskyy et al. (2009) [42] also revealed that activity of a mAChR-dependent 55-pS unitary current was absent in TRPC4-KO ileal myocytes, suggesting that TRPC4 corresponds to the muscarinic 70-pS channel described by Sakamoto et al. (2007) [23]. Alternatively, the TRPC6-mediated *mIcat* could also be activated by DAG, similar to the *mIcat* observed in PTX-treated wild-type mouse ileal myocytes [40,44], suggesting that the TRPC6 channel mediates the 120-pS unitary current activated through the M_3_ pathway [23].

Figure 1 presents a scheme of the three distinct signal transduction pathways linking mAChR subtypes to cationic channel activation and *mIcat* generation. The M_3_ pathway activates the brief opening mode of 70-pS (TRPC4) and 120-pS (TRPC6) channels via G_q/11_-PLC signalling. The lower conductance TRPC4 is regulated by the PLC substrate phosphatidylinositol 4,5-bisphosphate (PIP_2_), which stabilises the inactive conformation [45], while PIP_2_ depletion by M_3_ activity relieves channel inactivation, allowing transition of channel gating to the brief open mode. Alternatively, the higher conductance TRPC6 is opened by M_3_ via a direct action of DAG [40,44,45]. The M_2_ pathway activates the longer opening mode of TRPC4 and independently inhibits adenylyl cyclases via G_i/o_ proteins [36]. The M_2_/M_3_ pathway transmits distinct concurrent signals to TRPC4 channels, with the M_3_/G_q/11_/PLC branch providing a permissive signal for channel gating through PIP_2_ depletion and the M_2_/G_o_ branch allowing transition to a gating state with long openings and brief closings depending on activation strength of the receptor or G-protein [23,36,45,46]. Evidence has shown that the M_2_/M_3_ pathway, but not the separate M_2_ and M_3_ pathways, allows for potentiation of channel activation by Ca^2+^ [23], so this pathway is strengthened by M_3_/InsP_3_-dependent Ca^2+^ release. Whether the M_2_/M_3_ pathway can significantly stimulate InsP_3_/DAG formation and/or significantly inhibit cAMP accumulation remains unknown. A study in rabbit intestinal smooth muscle has suggested that G_o_ protein is not involved in mAChR-mediated adenylyl cyclase inhibition [24].

The M_2_/M_3_ pathway appears to involve the formation of a tight connection among M_2_, M_3_ and cationic channels via G-proteins G_o_ for the M_2_, and G_q/11_ for the M_3_ [17,31,47], a hypothesis recently tested using mAChR-mutant mice [39]. As mentioned previously, carbachol-evoked *mIcat* in ileal myocytes was markedly reduced by M_3_-KO due to the absence of activated G_q/11_ proteins. In M_3_-KO ileal myocytes, prostaglandin F_2α_ (PGF_2α_) was able to stimulate the G_q/11_/PLC/InsP_3_-induced Ca^2+^ release pathway as measured by Ca^2+^-activated K^+^ current activation, but it was unable to potentiate the reduced carbachol-evoked *mIcat*. Similar results were obtained using neuropeptide Y (NPY) in M_2_-KO myocytes, where carbachol-evoked *mIcat* was also reduced due to the absence of activated G_o_ proteins. NPY was reported to stimulate the G_i/o_/adenylyl cyclase system in intestinal smooth muscle [48]. However, it did not potentiate the reduced carbachol-evoked *mIcat* in M_2_-KO mice. If the G_o_ and G_q/11_ proteins involved in the M_2_/M_3_ pathway were activated by NPY and PGF_2α_, respectively, the reduced *mIcat* should have been increased toward the wild-type *mIcat*. However, there was no such potentiation, implying that the G-proteins (G_o_ and G_q/11_) involved in the M_2_/M_3_ pathway are specific for this signalling pathway and supporting segregation of the M_2_/M_3_ pathway by tight coupling among mAChRs (M_2_/M_3_), G proteins (G_o_/G_q/11_), cationic channels (TRPC4) and other elements.

From these findings arises an enigma. Previous studies on guinea-pig ileal myocytes reported that an anti-G_αq/11_ antibody did not significantly influence carbachol-evoked *mIcat* but did block carbachol-evoked K^+^ currents secondary to M_3_/G_q/11_/PLC/InsP_3_-mediated Ca^2+^ release [35,37]. In contrast to the anti-G_αq/11_ antibody, M_3_ receptor antagonists, the PLC inhibitor U-73122 and the G_q/11_ inhibitor peptide YM-254890 have been shown to inhibit both carbachol-evoked currents. Although the exact reason for this unexpected behaviour of the anti-G_αq/11_ antibody is unknown, a possible explanation is that the M_2_/M_3_ signalling complex is tight not only functionally but also structurally such that the associated G_q/11_ proteins are inaccessible to the antibody, while the G_q/11_ proteins involved in other signalling pathways such as the discrete M_3_ receptor pathway are accessible to the antibody. Indeed, Aslanoglou et al. (2015) [50] reported M_2_/M_3_ heteromers at the surface of cells stably expressing human wild-type M_2_ receptor and a Receptor Solely by Synthetic Ligand (RASSL) variant of the human M_3_ receptor. Therefore, M_2_/M_3_ receptor heterodimers may form a structural complex with G_o_ and G_q/11_, and this complex may then form specific associations with downstream signals that activate *mIcat*.

### 3.2. Regulation of K^+^ and Cl^−^ Channels

K^+^ channels are important targets for ACh and secondary messengers in gastrointestinal smooth muscles as they are the main regulators of the resting membrane potential and critical modulators of excitation kinetics. Muscarinic modulation of various types of K^+^ channels has been reported [12,30], including the opening of large conductance Ca^2+^-activated K^+^ (BK) channels secondary to intracellular Ca^2+^ release through the M_3_/G_q/11_/PLC/InsP_3_ signalling pathway [47,51]. Sakamoto et al. (2007) [23] measured BK currents evoked by carbachol in ileal myocytes isolated from mAChR-mutant and wild-type mice at a holding potential of 0 mV, near the reversal potential of *mIcat*. At this potential, cells exhibited spontaneous transient outward currents (STOCs) reflecting the opening of a population of BK channels in response to sporadic localised Ca^2+^ release from internal stores [52]. Application of maximally effective carbachol (100 µM) to wild-type cells produced a large transient BK current followed by abolition of STOCs due to depletion of Ca^2+^ stores. Current kinetics were similar in M_2_-KO cells, while M_3_-KO and M_2_/M_3_-KO cells exhibited no such carbachol-activated outward current. These results indicate that intracellular Ca^2+^ release required for BK current activation is triggered exclusively by M_3_ receptor-induced InsP_3_ generation in gut smooth muscle. In addition to BK channel activation, muscarinic stimulation is reported to suppress BK channel activity through a PTX-sensitive mechanism, suggesting mediation by M_2_ receptors, in canine gut and equine airway myocytes [53,54]. However, we observed that carbachol evoked neither BK currents nor significant changes in the size and shape of STOCs in M_3_-KO mouse ileal myocytes (unpublished data), indicating a lack of M_2_ receptor coupling to BK channels, at least in this cell type.

Visceral smooth muscle cells also expressed ATP-sensitive K^+^ (K_ATP_) channels, which are inhibited by intracellular ATP or glibenclamide and activated by the K^+^ channel openers such as cromakalim. Muscarinic suppression of K_ATP_ channels has been reported in urinary bladder [55], oesophageal [56] and tracheal smooth muscles [57]. Pharmacological studies suggest that channel suppression is mediated by M_3_ receptors and involves PKC activity. Recently, Wang et al. (2018) [58] found that carbachol (100 µM) inhibited cromakalim-activated K_ATP_ current to a similar extent in ileal myocytes from wild-type and M_2_-KO mice but had no effect on ileal myocytes from M_3_-KO and M_2_/M_3_-double-KO mice, suggesting that this muscarinic suppression of K_ATP_ channels is mediated primarily by M_3_. Further, muscarinic suppression of the K_ATP_ current was blocked by the G_q/11_ inhibitor YM-254890 and the PLC inhibitor U73122, but not by the PKC inhibitor calphostin C, suggesting the involvement of a G_q/11_/PLC-dependent but PKC-independent signalling pathway. Wild-type ileal myocytes responded to cromakalim by hyperpolarisation and to glibenclamide by depolarisation, implying that K_ATP_ channels contribute significantly to the muscarinic regulation of cellular excitability and contraction.

Chloride channels are expressed in many smooth muscles and contribute to depolarisation, as the Cl^−^ equilibrium potential is well above the resting potential [30]. Like BK channels, chloride channel opening is triggered by intracellular Ca^2+^ release. In tracheal smooth muscle, muscarinic agonist-evoked Cl^−^ currents were blocked by M_3_ receptor antagonists or anti-G_q/11_ antibody [59,60]. In wild-type mouse ileal myocytes, carbachol (100 µM) evoked an inward current consisting of an initial rapid peak followed by a smaller sustained phase, and the initial rapid current was mediated in part by opening of Ca^2+^-activated Cl^−^ channels [40].

### 3.3. Inhibition of Voltage-Dependent Ca^2+^ Channels

Voltage-dependent Ca^2+^ channels (VDCCs) are the predominant pathway for Ca^2+^ entry into smooth muscle cells during depolarisation. Surprisingly, while mACh receptors can depolarise smooth muscle cells, receptor activation generally inhibits VDCC activity and suppresses the inward Ca^2+^ current (I_Ca_) [12]. This effect may constitute a negative feedback mechanism to prevent cytosolic Ca^2+^ overload during muscarinic depolarisation. In guinea-pig ileal myocytes, carbachol suppressed I_Ca_ in a biphasic manner, with an initial rapid suppression lasting ~10 s followed by a more sustained suppression subsiding slowly only after agonist removal [61,62]. The initial transient suppression reflects Ca^2+^-induced channel inactivation by the M_3_/G_q/11_/PLC/InsP_3_ pathway [61,62,63], while the mechanisms underlying the sustained phase are less clear. One group reported that both phases were insensitive to PTX, suggesting mediation via M_3_-G_q/11_ [62], whereas another reported that the sustained phase was sensitive to PTX and thus potentially mediated via M_2_-G_i/o_ [19]. These contradictory results underscore the importance of experimental conditions (such as voltage clamp modality and pipette solution) for I_Ca_ measurements and for examining the effects of various modulators.

Tanahashi et al. (2009) [49] attempted to link specific mAChR subtypes/G-protein combinations to carbachol-induced I_Ca_ inhibition in mouse ileal myocytes using mAChR-KO mice and the pharmacological tools PTX and YM-254890. In wild-type cells, carbachol caused a biphasic inhibition of I_Ca_, as described for guinea-pig ileal I_Ca_ (see above). In contrast, M_2_-KO cells showed a marked reduction in the sustained inhibition but no deficit in the initial transient inhibition, whereas M_3_-KO cells were deficient in the initial transient inhibition and displayed a marked reduction in the later sustained inhibition and no I_Ca_ inhibition occurred in the absence of M_2_ and M_3_ receptors. Accordingly, the muscarinic inhibition of I_Ca_ is mediated by both M_2_ and M_3_ receptors, but not other mAChR subtypes. The M_2_-mediated suppression in M_3_-KO ileal myocytes was abolished by PTX but unaffected by the G_q/11_ blocker YM-254890, while M_3_-mediated sustained suppression in M_2_-KO myocytes was unaffected by PTX but blocked by YM-254890. The magnitude of sustained suppression (% inhibition of I_Ca_) by carbachol in wild-type myocytes was much greater than the sum of M_2_- and M_3_-mediated inhibition, indicating that a considerable part of the inhibition in wild-type cells (~56%) requires co-activation of M_2_ and M_3_ receptors. Moreover, the sustained inhibition in wild-type cells was profoundly reduced by PTX or YM-254890, and the residual inhibition closely resembled M_3_- and M_2_-mediated inhibition, respectively. When [Ca^2+^]_i_ was strongly buffered by intracellular EGTA perfusion, M_2_- and M_3_-mediated inhibition remained unaffected, but carbachol-induced sustained inhibition in wild-type myocytes was markedly reduced, and the remaining I_Ca_ component resembled the sum of M_2_- and M_3_-mediated inhibition.

Taken together, these results indicate that M_2_ receptors mediate I_Ca_ suppression via G_i/o_ proteins in mouse ileal myocytes, while M_3_ receptors mediate both fast transient and slow sustained I_Ca_ suppressions via G_q/11_, and another pathway involving M_2_–M_3_ interaction is the major contributor to sustained carbachol-induced I_Ca_ suppression. This M_2_/M_3_ synergistic pathway, unlike the separate M_2_ and M_3_ pathways, involves a process where Ca^2+^ has permissive and/or potentiating action(s) on carbachol-induced sustained I_Ca_ suppression. These three distinct muscarinic pathways for I_Ca_ suppression show the typical features of *mIcat* activation (see Figure 1). Therefore, it is plausible that all three converge on both TRPC4/6 cationic channels and VDCCs. Activation of the former triggers depolarisation and Ca^2+^ entry via VDCCs, while the latter acts as a feedback brake to prevent cytosolic Ca^2+^ overload during depolarisation.

In contrast to I_Ca_ suppression by mAChRs, Jin et al. (2002) [64] reported that the muscarinic agonist methacholine enhanced I_Ca_ in rabbit colonic myocytes under conditions where M_3_ signalling was disrupted by an M_3_ receptor antagonist or anti-G_αq_ antibody, suggesting a possible pathway linking M_2_-receptor activation to I_Ca_ enhancement. However, in M_3_-KO mouse ileal myocytes, carbachol caused no potentiation of I_Ca_ but rather induced phasic suppression. One possible explanation for this discrepancy in I_Ca_ potentiation by M_2_ signalling is that both inhibitory and facilitatory pathways operate in parallel, with the balance modified by currently unknown factors.

Studies on ileal myocytes from mAChR-KO mice indicate that the M_2_ subtype participates in regulation of TRPC4 channels and VDCCs, but not TRPC6, BK, K_ATP_ or Cl^−^ channels, whereas the M_3_ subtype appears to regulate all of these ionic channels.

## 4. Depolarisation

Numerous studies have examined membrane potential changes induced by muscarinic agonists and by enteric nerve stimulation in mAChR-KO mice. In general, results are in agreement with macroscopic *mIcat* and single channel current studies.

**Figure 2:**
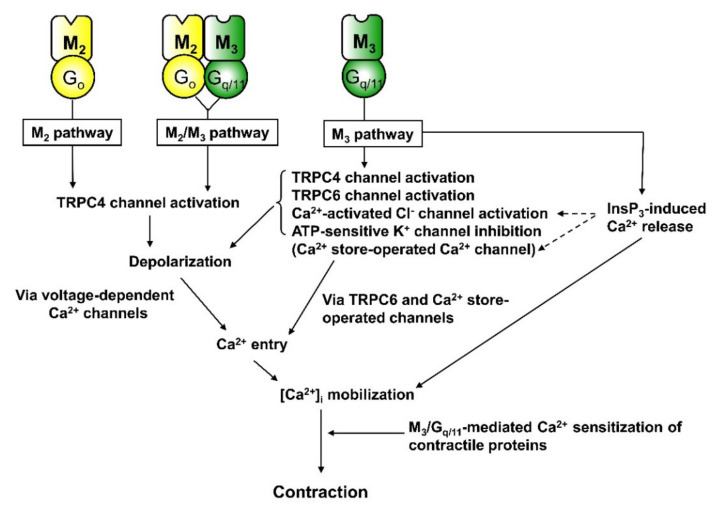
Signal transduction mechanisms underlying muscarinic contraction of mouse intestinal smooth muscle. The M_3_ pathway activates multiple intracellular Ca^2+^ mobilisation events, including Ca^2+^ influx via VDCCs and voltage-independent Ca^2+^-permeable channels and intracellular Ca^2+^ release. Voltage-dependent Ca^2+^ influx is initiated by depolarisation from opening of TRPC4 and TRPC6 cationic channels, opening of Ca^2+^-activated Cl^−^ channels and inhibition of K_ATP_ channels, while voltage-independent Ca^2+^ entry is mediated by opening of TRPC6 cationic channels and Ca^2+^ store-operated Ca^2+^ channels. In addition to Ca^2+^ mobilisation, Ca^2+^ sensitisation of contractile proteins is elicited, thereby increasing the efficiency of contraction–[Ca^2+^]_i_ coupling. The M_2_ or M_2_/M_3_ pathways induce contraction through a simple Ca^2+^ mobilisation mechanism in which Ca^2+^ entry via VDCCs is activated by TRPC4 channel-induced depolarisation. These pathways play a major role in mediating muscarinic contraction, with the M_2_/M_3_ pathway making a relatively greater contribution at low agonist concentrations. The M_2_ pathway also induces contraction indirectly by inhibiting cAMP-dependent relaxation in response to adenylyl cyclase-activating agonists.

Changes in membrane potential produced by carbachol were recorded in ileal myocytes from mAChR-KO and wild-type mice using the nystatin-perforated patch-clamp technique [23] under physiological ionic conditions (high extracellular Na^+^ and intracellular K^+^), where the resting membrane potentials were similar among the different mouse strains. The wild-type cells responded to 0.1–0.3 µM carbachol with a moderate depolarisation of 10–20 mV and to higher concentrations (>1 µM) by a depolarisation of 40–50 mV, reaching the equilibrium potential for muscarinic depolarisation (−10 mV) [65,66]. In M_2_-KO cells, 0.1 µM of carbachol was without effect, but 1 µM was equipotent to the effect of 0.1 µM carbachol on wild-type cells and 30–100 µM evoked a full depolarisation, indicating that the M_2_ receptor contributes substantially to depolarisation at low agonist concentrations. In M_3_-KO cells, 1–3 µM carbachol caused only a slight depolarisation, and even 30–100 µM evoked a depolarisation of up to only 10 mV. In the M_2_/M_3_ double-KO cells, carbachol induced no depolarisation, but PGF_2α_ caused a prominent depolarisation. Taken together, these observations suggest that the three muscarinic pathways depolarise the membrane with the following rank order of efficacy: M_2_/M_3_ > M_3_ >> M_2_ pathway. Both M_2_/M_3_ and M_2_ pathways lead to depolarisation only through activation of TRPC4 channels, while the M_3_ pathway depolarises the membrane through a combination of (at least) TRPC4, TRPC6 and Cl^−^ channel activation and K_ATP_ channel inhibition (see Figure 2).

Excitatory junction potentials (EJPs) evoked by enteric nerve stimulation [67] have also been measured in smooth muscle cells from mAChR-KO mice. Matsuyama et al. (2013) [67] compared EJPs evoked by electrical field stimulation (EFS) in longitudinal muscle strips from the ileum of mAChR-KO and wild-type mice using intracellular microelectrodes and found that responses in the wild-type strips were strongly inhibited, but not abolished, by PTX and by the cationic channel blocker SK&F96365 [68]. Alternatively, cholinergic EJPs evoked in M_2_-KO strips were only 20%–30% of the wild-type amplitude and markedly reduced but not abolished by SK&F96365. In contrast, no EJP was elicited in M_3_-KO or M_2_/M_3_ double-KO strips. Taken together, these results clearly demonstrate that both M_2_ and M_3_ receptors contribute to cholinergic EJPs in ileal myocytes and further suggest that wild-type cholinergic EJPs occur through both M_2_/M_3_ and M_3_ pathways with a greater contribution from the M_2_/M_3_ pathway. In contrast, M_2_ receptors alone do not elicit large EJPs. Note, however, that the application of drugs to prevent muscle contraction (nifedipine and papaverine) or simultaneous inhibitory neurotransmission (L-NAME and guanethidine) may influence these responses.

## 5. Ca^2+^ Sensitisation of Contraction

Muscarinic receptors control contractile tension in smooth muscle primarily by inducing elevations in [Ca^2+^]_i_. In addition, however, mAChRs can also regulate smooth muscle contraction by enhancing the Ca^2+^ sensitivity of contractile proteins. This mAChR-mediated Ca^2+^ sensitisation occurs through two distinct pathways involving the small G-protein Rho/Rho kinase and the DAG target PKC, respectively [69,70]. Both pathways may be initiated by M_3_ receptors [28], and possible positive or negative regulation of Ca^2+^ sensitivity by M_2_ receptors via other kinases has also been suggested [28,71,72].

Suguro et al. (2010) [73] characterised the mAChR subtypes mediating carbachol-induced Ca^2+^ sensitisation by comparing the [Ca^2+^]_i_ (pCa)–tension relationship among α-toxin-permeabilised ileal muscle strips from mAChR-KO and wild-type mice. In strips from wild-type mice, isometric tension responses to Ca^2+^ applied cumulatively (pCa 7.0–5.0) were increased by carbachol (100 µM) as indicated by shifts in both the 50% effective concentration (EC_50_) and the maximum response (E_max_). The M_2_-KO strips exhibited similar changes, while M_3_-KO and M_2_/M_3_ double-KO strips showed little or no change in pCa–tension curves. As expected from mAChR-mediation, the direct G-protein activator GTPγS altered both EC_50_ and E_max_ in all KO and wild-type strips without additional stimulation, and carbachol-induced Ca^2+^ sensitisation in the wild-type and M_2_-KO strips was totally blocked by the G_q/11_ inhibitor YM-254890. These results provide strong evidence that mAChR-mediated Ca^2+^ sensitisation of contractility in intestinal smooth muscle occurs through coupling of M_3_ receptors to G_q/11_ proteins, consistent with the pharmacological study of Murthy et al. (2003) [28], while there is currently no evidence for involvement of M_2_ receptors in regulation of myofilament Ca^2+^ sensitivity.

## 6. Heterologous Desensitisation of Contraction

It is well known that prolonged exposure of gastrointestinal smooth muscle to a muscarinic agonist decreases the contractile response evoked by subsequent exposure to a muscarinic agonist or other spasmogen such as PGF_2α_ and histamine. In guinea-pig ileum, ACh-induced desensitisation of the contractile response to histamine was prevented by disruption of M_2_/G_i/o_ signalling with PTX or by M_3_ receptor inactivation with 4-DAMP mustard [74]. This and other pharmacological results suggest that this mAChR-mediated heterologous desensitisation depends on activation of both M_2_ and M_3_ receptors. Griffin et al. (2004) [75] investigated the mAChR subtypes mediating heterologous desensitisation in the isolated ileum using mAChR-KO and wild-type mice. Prolonged ACh treatment of wild-type mouse ileum (30 µM for 20 min) desensitised the subsequent contractile response to both PGF_2α_ and oxotremorine-M as indicated by significant increases in EC_50_ measured 5 min after ACh wash-out. The desensitisation to PGF_2α_ was prevented by either M_2_-KO or M_3_-KO and that to oxotremorine-M by M_2_-KO. These results indicate that muscarinic agonist-induced heterologous desensitisation requires activation of both M_2_ and M_3_ receptors and that activation of either receptor by itself is insufficient, in accordance with a previous study in guinea-pig ileum [74].

The requirement of M_2_ and M_3_ receptor co-activation for heterologous desensitisation further hints at the underlying mechanism. As described earlier, synergy between M_2_ and M_3_ receptors (or M_2_/M_3_ pathway activation) results in extensive opening of TRPC4 non-selective cation channels. This may result in intracellular Na^+^ accumulation and loss of intracellular K^+^, thereby accelerating transmembrane Na^+^-K^+^ pump activity. The resulting hyperpolarisation from increased pumping activity (as the exchanger expels 3 Na^+^ for every 2 K^+^ pumped back in) may reduce the sensitivity to depolarising agents [76]. The inhibition of VDCC activity, which occurs through M_2_/M_3_ interaction, may also participate in heterologous desensitisation since it has been shown to subside slowly after cessation of mAChR stimulation [19].

## 7. Smooth Muscle Contraction

Pharmacological evidence has shown that the less abundant M_3_ receptor is largely responsible for mediating direct contractile responses to applied muscarinic agonists or neurogenic ACh [1,9], while most (but not all) studies have found no or little evidence for involvement of the more abundant M_2_ receptor in contractile responses [16,77,78]. However, M_2_ receptor activation induces contraction indirectly by preventing the cAMP-dependent relaxation effects of forskolin and isoprenaline in the presence of a stimulatory receptor agonist such as histamine [79]. The availability of mAChR-KO mice has promoted studies to definitively determine which mAChR subtype(s) mediate direct or indirect contraction and to elucidate muscarinic contractile mechanisms in gastrointestinal smooth muscle.

### 7.1. Contraction Evoked by Applied Muscarinic Agonists

In many studies, carbachol or oxotremorine-M was applied with cumulative or single dose application protocols, and concentration–response curves for the agonist-evoked contraction were constructed to yield E_max_ and EC_50_. The contributions of individual mAChR subtypes in contraction have been characterised based on concentration–response changes in KO mice. Table 1 presents E_max_ values for different mutant strains expressed relative to the corresponding wild-type E_max_ garnered either from published values or extracted from concentration–response curves, while Table 2 presents published pEC_50_ values (negative logarithm of EC_50_). Parameters obtained from other visceral smooth muscles are also included in Table 1 and Table 2.

Knockout of M_3_ receptors markedly reduced E_max_ in the stomach (fundus, antrum and body), ileum and colon (proximal and distal), with estimated E_max_ values ranging from 21% to 66% of the wild-type E_max_ among different tissues and studies of the same tissue. Similar effects of M_3_-KO were also found in other visceral smooth muscle tissues, with reductions to 5–15% of the wild-type E_max_ in the urinary bladder, 44% in the trachea, 21% in the gallbladder and complete elimination in the uterus. Alternatively, there were few significant changes in pEC_50_ or agonist potency except for a two-fold increase in the ileum and stomach bodies and a two-fold decrease in the urinary bladder (Table 2).

Unlike M_3_ receptor KO, M_2_ receptor KO did generally not markedly alter E_max_ (65–86% of wild-type E_max_ in the colon, trachea, urinary bladder and uterus; Table 1). In line with the results in the urinary bladder, a highly selective but not commercially available M_2_ antagonist THRX-182087 had little effect on the amplitude of the CCh-induced contractions in the rat urinary bladder [94]. Alternatively, a significant decrease in pEC_50_ was observed, with most agonist concentration–response curves demonstrating a 2- to 3-fold decrease in pEC_50_ in M_2_-KO mice (Table 2). Further, no tissues from M_2_/M_3_ double-KO mice exhibited detectable contraction, and gastrointestinal tissues actually showed relaxation in response to the agonist (Table 1). Therefore, it is highly probable that M_2_ and M_3_ receptors, but not other mAChR subtypes, mediate contraction in all visceral smooth muscle studied. Indeed, M_4_-KO had little effect on E_max_ and pEC_50_ for carbachol in the stomach fundus, urinary bladder and trachea [80]. In contrast, M_4_-KO in the gallbladder did cause a dextral shift in the carbachol concentration–response curve, suggesting that the M_4_ receptor may provide a signal for optimal carbachol potency in this particular tissue (see Figure 1 in Stengel and Cohen, 2002 [91]). However, the gallbladder contractile response to carbachol is unique in that it is associated with release of a cyclooxygenase (COX) product [91].

The kinetics of contraction evoked by a single dose of carbachol differ considerably between M_2_-KO and M_3_-KO mice, features particularly apparent in the stomach antrum and fundus, longitudinal muscle of the ileum and both distal and proximal colon [82,87,90]. Specifically, contraction in M_3_-KO tissues (M_2_-mediated) exhibited a phasic form characterised by an initial rapid peak in tension followed by a gradual decline even in the continued presence of the agonist, while contraction in M_2_-KO tissues (M_3_-mediated) exhibited a rapid rise to peak and persistence in the continued presence of the agonist. The later form resembled that of wild-type tissues, consistent with the documented predominance of M_3_ in mediating the contractile response to carbachol. As mentioned above, although pharmacological studies have found no or little evidence for involvement of the M_2_ receptor in contractile responses [16,77,78], these studies using the muscarinic receptor subtype-KO mice revealed that the M_2_ can directly induce contraction in gastrointestinal smooth muscles.

A contribution of M_1_ receptor-mediated NO release has been suggested in the relaxation of gastrointestinal smooth muscle [95], as the phasic carbachol-induced contraction in M_3_-KO colon was changed to a sustained contraction by the NOS inhibitor L-NAME or TTX [90]. A similar mechanism may also contribute to carbachol-evoked relaxation in M_2_/M_3_ double-KO gastrointestinal tissue [82]. In addition, Stengel and Cohen (2003) [96] reported that M_3_ receptor KO unmasked neurogenic NO-dependent relaxation evoked by carbachol in the mouse stomach fundus, resulting in a bell-shaped concentration–response curve for the contractile effect of carbachol. Thus, NO released from nerve endings by stimulation of muscarinic receptors other than M_2_ and M_3_ receptors may affect the muscarinic contractions in gastrointestinal smooth muscles.

The use of mAChR-mutant mice has revealed that muscarinic agonist-induced contraction in visceral smooth muscles is mediated predominantly by a combination of M_2_ and M_3_ receptors, with a greater contribution of M_3_, generally consistent with previous pharmacological studies. It has also been demonstrated that activation of M_2_ receptors can evoke a direct contraction in most M_3_-KO visceral tissues studied (except for the gallbladder, in which muscarinic contraction involves prostaglandins), whereas pharmacological studies have found no or little evidence for involvement of the M_2_ receptor in contractile responses (see above). The E_max_ of carbachol varies considerably among different tissues of M_3_-KO mice (ranking: stomach, ileum and colon = trachea < urinary bladder ≪ uterus) (see Table 1). This variation in M_2_-dependent E_max_ may reflect different coupling efficiencies between M_2_ receptors and downstream contractile mechanisms, but could also indicate distinct M_2_-mediated contractile mechanisms. In the uterus, for example, M_2_ receptors have no or little contractile activity in M_3_-KO mice, but can enhance M_3_-mediated contraction in the wild-type uterus [93].

As expected, PTX treatment markedly depressed carbachol-induced contraction in the M_3_-KO mouse ileum, but had no effect on the M_2_-KO mouse ileum [87]. The involvement of M_2_/G_i/o_ signalling in the contraction of the wild-type ileum was then characterised using the toxin. Changes in the concentration–response curve to carbachol by PTX treatment indicated a significant contribution of M_2_/G_i/o_ signalling only at relatively low agonist concentrations (by ~70% at 0.1 µM but only 40% at 1 µM and no reduction at 10 µM carbachol), while M_3_/G_q/11_ signalling was completely dominant at higher agonist concentrations. These findings and those from the aforementioned electrophysiological studies indicate that M_2_/G_i/o_ signalling, probably via a synergy with M_3_/G_q/11_ signalling, may produce contraction at relatively low agonist concentrations in intact gastrointestinal smooth muscle co-expressing M_2_ and M_3_ receptors.

### 7.2. Cholinergic Nerve-Mediated Contraction

Studies using mAChR-KO mice have also helped define the mAChR subtypes mediating contraction in gastrointestinal smooth muscles evoked by cholinergic nerve stimulation [82,88,97]. In the wild-type ileum, EFS evoked a rapid phasic contraction followed by a sustained contraction lasting for 2–8 min after cessation. The initial contraction was arrested by the anticholinergic atropine, confirming cholinergic nerve dependence. Cholinergic contractions evoked by EFS at 5 to 50 Hz were also significantly reduced by M_2_- or M_3_-KO. In both tissues, the residual contraction strength was ~80% of the wild-type control regardless of stimulus frequency [97]. Similar results were also obtained by Takeuchi et al. (2007) [88] under similar experimental conditions, with 1–10 Hz EFS evoking contractions in the ileum from M_2_-KO mice resembling those in the wild-type and corresponding contractions in M_3_-KO mice reaching 70 to 80% of wild-type contraction strength. Kitazawa et al. (2007) [82] reported that the cholinergic contractions in the gastric fundus and antrum induced by 32 Hz stimulation were reduced by both M_2_-KO (71% of wild-type response in the fundus, 75% in the antrum) and M_3_-KO (54% of wild-type in the fundus and 54% in the antrum), while M_2_/M_3_ double-KO completely eliminated EFS-evoked cholinergic contraction in the stomach and ileum. Collectively, these results clearly demonstrate that cholinergic nerve-mediated contractions in gastrointestinal smooth muscle are mediated by a combination of M_2_ and M_3_ receptors, with M_3_ predominance.

The EFS-evoked cholinergic contractions in the M_3_-KO ileum were completely arrested by PTX treatment, indicating that M_2_/G_i/o_ signalling activity is essential in the absence of M_3_ receptors. In the wild-type ileum, EFS-evoked contractions at 10–50 Hz were depressed by 20–30% by PTX treatment, consistent with mediation by a combination of M_2_ and M_3_ receptors. Curiously, EFS-evoked contractions at 2 Hz were increased and those at 5 Hz unchanged by PTX. To explain these observations, Unno et al. (2006) [97] speculated that the loss of M_2_ signalling by PTX treatment was overcome or balanced by increased M_3_ receptor signalling, probably owing to suppressed presynaptic autoinhibition of ACh release mediated by M_2_/G_i/o_ [20].

**Figure 3:**
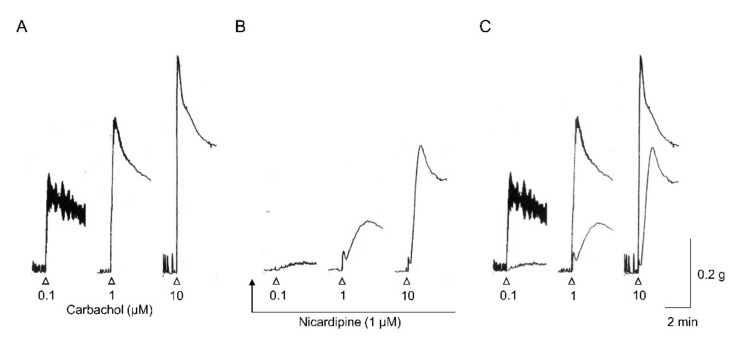
Effects of nicardipine on carbachol-evoked contractions in a wild-type mouse ileal longitudinal muscle strip. Carbachol was applied for 3 min at the indicated concentrations before (**A**) and after nicardipine treatment to block VDCCs (**B**). In (**C**), the recording traces in A and B are superimposed. Note that when muscarinic stimulation is weak, contraction is relatively more dependent on voltage-dependent Ca^2+^ entry and is more sensitive to PTX. This finding indicates that the M_2_/M_3_ (and M_2_) pathway has a major role in mediating the contractile response to weak muscarinic stimulation. When receptor stimulation is stronger, the M_3_ pathway predominates by activating multiple mechanisms for Ca^2+^ mobilisation as well as myofilament Ca^2+^ sensitisation.

Under conditions where non-adrenergic neural inputs were pharmacologically minimised, contractions evoked by 20 Hz, 5 s EFS trains in the wild-type mouse ileum were almost abolished by atropine (93% reduction) or the VDCC blocker nifedipine (95% reduction) (Komori, Tanahashi, Matsuyama, Unno; unpublished data). These results indicate that cholinergic contractions in the mouse ileum depend largely on Ca^2+^ entry via VDCCs, similar to contraction evoked by low-dose muscarinic agonists (see Figure 3). 

Tsvilovskyy et al. (2009) [42] also reported a 64% reduction in cholinergic contraction among TRPC4-deficient mice compared to wild-type controls. However, PTX treatment reduced these cholinergic contractions in the wild-type ileum by only ~30%, an inconsistency that remains to be clarified.

Interstitial cells of Cajal (ICCs) distributed within the smooth muscle layer have been implicated in enteric neurotransmission based on their close proximity to the varicosities of enteric motor neurons and expression of receptors for enteric neurotransmitters (see Sanders et al. 2014 [98]). Quantitative PCR revealed expression of both M_2_ and M_3_ muscarinic receptor transcripts in intramuscular ICCs (ICC-IMs) of the murine colon [99] and deep muscle plexus ICCs (ICC-DMPs) of the murine small intestine [100]. In contrast to smooth muscles (see above), *Chrm3* (M_3_) expression was higher than *Chrm2* (M_2_) expression in ICC-IMs and ICC-DMPs. Drumm et al. (2020) reported that EFS increased Ca^2+^ transients in colonic ICC-IMs, a response abolished by atropine and the M_3_ receptor antagonist DAU 5884 but not the M_2_ antagonist AF-DX 116. Furthermore, pharmacological inhibition of the anoctamin-1 (ANO1) Ca^2+^-activated Cl^−^ channel expressed selectively in ICCs suppressed EFS-induced cholinergic contractions in the murine colon. These observations suggest that enhancement of Ca^2+^ transients by acetylcholine stimulation of M_3_ receptors activates ANO1 channels in colon ICC-IMs resulting in membrane depolarization. The depolarization can be conducted to adjacent smooth muscle cells through gap junctions, leading to excitation of the smooth muscle cells. Alternatively, the physiological functions of M_2_ receptors expressed by ICC are still unknown. Groneberg et al. (2006) [101] suggested parallel neurotransmission of enteric NO to ICCs and smooth muscle cells in the murine fundus. Thus, it will be of particular interest to reveal the effects of each muscarinic receptor subtype expressed by ICCs on muscarinic contraction and to compare the underlying signal transduction pathways with those of smooth muscle cells. Mutant mice generated with mAChR subtype-specific KO (e.g., Gautam et al. 2006 [102]) in ICCs and smooth muscle cells [101] using the Cre/LoxP recombination system should be useful to address these issues.

### 7.3. Indirect Contraction by Inhibiting Cyclic AMP-Dependent Relaxation

Pharmacological studies of visceral smooth muscles across species have demonstrated that M_2_ receptors contribute to contraction by inhibiting relaxation caused by agents that increase cAMP [9,15]. To establish an indirect role of M_2_ receptors in smooth muscle contraction, Matsui et al. (2003) [86] investigated the ability of the adenylyl cyclase activators forskolin and isoprenaline to inhibit oxotremorine-M-induced contraction in smooth muscles of the the ileum, urinary bladder and trachea from wild-type and M_2_-KO mice. The relaxant effects of forskolin against oxotremorine-M contraction were greatly increased in all three tissues from M_2_-KO mice compared to wild-type controls. Under similar conditions, the relaxant effects of isoprenaline were also enhanced in the ileum and urinary bladder from M_2_-KO mice. These results strongly suggest that M_2_ receptors suppress cAMP-dependent relaxation by inhibiting adenylyl cyclases [103]. In the trachea, however, no difference in isoprenaline-induced relaxant effect was found between wild-type and M_2_-KO mice. A similar conclusion was reached in pharmacological studies of guinea-pig and bovine trachea [104,105]. The difference in response between the trachea and other smooth muscle tissues remains to be explained [15]. In accord with agonist treatment, isoprenaline also induced a relaxant effect on the cholinergic contraction evoked by EFS in the urinary bladder from wild-type mice [106], while this effect was reduced by M_2_-KO mice, suggesting that M_2_ receptors indirectly promote contraction of gastrointestinal smooth muscle in response to cholinergic nerve stimulation.

Collectively, M_2_ receptors appear to facilitate contractility of smooth muscles by counteracting the relaxant effects of agents that increase intracellular cAMP. In visceral smooth muscle, the cholinergic muscarinic system serves to suppress sympathetically mediated relaxation through M_2_-mediated adenylyl cyclase inhibition.

### 7.4. Muscarinic Contractile Mechanisms in Mouse Intestinal Smooth Muscle

The signal transduction pathways linking mAChR activation to contraction of mouse intestinal smooth muscle are summarised in Figure 2. Unno et al. (2005) [87] characterised Ca^2+^ sources associated with pure M_2_- and pure M_3_-mediated contractions in ileal longitudinal muscle of M_2_-KO and M_3_-KO mice using VDCC blockers such as nicardipine and nifedipine, depolarising high-K^+^ medium for depolarization block of VDCCs and Ca^2+^-free medium for block of Ca^2+^ influxes. The pure M_2_-mediated contraction in M_3_-KO muscle strips was dependent exclusively on Ca^2+^ entry through VDCCs, especially influx associated with action potentials initiated or enhanced by TRPC4 channel activation (see Figure 1). On the other hand, the pure M_3_-mediated contraction in M_2_-KO muscle strips involved multiple Ca^2+^ mobilisation mechanisms, including both voltage-dependent and -independent Ca^2+^ entry and InsP_3_-induced Ca^2+^ release from intracellular stores. Voltage-dependent Ca^2+^ entry appears to be mediated by action potentials and sustained depolarisation caused by concomitant opening of TRPC4, TRPC6 and Cl^−^ channels and closing of K_ATP_ channels, while voltage-independent Ca^2+^ entry appears to be mediated directly by influx through TRPC6 channels and other Ca^2+^-permeable channels activated by Ca^2+^ store depletion [107]. In addition to Ca^2+^ mobilisation, pure M_3_ contraction is also associated with Ca^2+^ sensitisation of contractility, which serves to increase contraction efficiency.

As mentioned above, the M_2_ receptor has direct, potent contractile activity in the mouse ileum. Griffin et al. (2009) [89] explored whether such M_2_ activity was shared by the guinea-pig ileum using the irreversible M_3_ receptor antagonist 4-DAMP mustard. First, the M_3_ selectivity of the antagonist was confirmed in the ileum from mAChR-mutant and wild-type mice. Following 4-DAMP mustard treatment, the concentration–response curve for oxotremorine-M-evoked contraction in the wild-type ileum closely resembled that in the M_3_-KO ileum as indicated by similar pEC_50_ and E_max_ values and inhibition by common muscarinic antagonists. Thus, 4-DAMP mustard appears to inactivate M_3_ receptors selectively. Similar experiments were then conducted on the guinea-pig ileum. Following mustard treatment, the contractile response to oxotremorine-M exhibited a competitive antagonism profile consistent with an M_3_ response, suggesting that the guinea-pig ileum lacks a direct, potent M_2_-contractile component. It should be noted again that TRPC4 channel activation is the primary mechanism underlying M_2_-mediated contraction in the mouse ileum (see Figure 2), and there is abundant evidence that the gating properties and activation mechanism of TRPC4 channels are shared by both the mouse and guinea-pig ileum [23,36,40,45,46,108]. Contraction mediated by M_2_ receptors depends entirely on voltage-dependent Ca^2+^ entry, especially influx associated with action potentials, but does not involve Ca^2+^ sensitisation of contractility, so there is a tendency for attenuation of contraction by physiological relaxation factors such as neuronal NO and by deterioration of action potential amplitude during prolonged higher-frequency mAChR stimulation [82,87,90,96]. Hence, before it is established that the guinea-pig ileum lacks the direct, potent M_2_ contractile component, further study is needed to test for the contributions of factors likely to diminish M_2_-mediated contraction.

In addition to independent M_2_ and M_3_ pathways, the M_2_/M_3_ pathway also functions in the contraction of the wild-type mouse ileum. This pathway leads to TRPC4 channel activation and *mIcat* generation, which in turn depolarises the cell and stimulates voltage-dependent Ca^2+^ influx through VDCCs, similar to contraction induced by the M_2_ pathway. However, this pathway is much more potent than the M_2_ pathway at activating TRPC4 channels and generating *mIcat*. Effects of nicardipine, a VDCC blocker, were examined on carbachol-evoked contraction in isolated longitudinal muscle strips from the wild-type mouse ileum. As shown in Figure 3 (Komori, Tanahashi, Matsuyama, Unno; unpublished data), nicardipine abolished contraction in response to low concentrations of carbachol (0.1 µM), indicating that this contractile response is mediated primarily by voltage-dependent Ca^2+^ entry. Alternatively, nicardipine blockade was only moderate at 1 µM and markedly reduced at 10 µM. This agonist dose-dependence resembles that of PTX treatment, which blocked contraction substantially at 0.1 µM carbachol (~70%) and moderately at 1 µM (40%) but had no effect at 10 µM carbachol [87]. In addition, deletion of InsP_3_ receptor 1 dramatically reduced contraction induced by 10 µM carbachol in colonic circular smooth muscles, suggesting the importance of InsP_3_-dependent Ca^2+^ mobilization [109]. Collectively, these findings suggest that the M_2_/M_3_ pathway (and the M_2_ pathway) are major inducers of contraction under weak mAChR stimulation, while the M_3_ pathway predominates under stronger mAChR stimulation.

These predicted muscarinic pathways for contraction, especially the M_2_/M_3_ and M_3_ pathways, are analogous to those in a model proposed by Sawyer and Ehlert (1999) [16] who studied the interaction between M_2_ and M_3_ receptors in contraction of the guinea-pig colon. In their model (Model II), occupation of M_3_ receptors activates two parallel signalling pathways, one causing simple M_3_-mediated contraction and one with no effect alone but activated in the presence of occupied M_2_ receptors (a conditional pathway).

## 8. In Vivo and In Vitro Gastrointestinal Motility

Ultimately, the relevance of these muscarinic pathways depends on their effects on gastrointestinal motility in vivo. Yamada et al. (2001) [110] examined the effects of M_3_-KO on gut smooth muscle activity in vivo by administering intragastric charcoal and monitoring the distance travelled, which revealed no effect on gut motor activity. Kitazawa et al. (2007) [82] also found that estimated gastric emptying rates as assessed by measuring the weight of food intake relative to that in the stomach 30 min later were similar between M_2_/M_3_ double-KO and wild-type mice, again indicating no significant change in gastric motility in the absence of M_2_ and M_3_ receptors. These results are seemingly at odds with in vivo pharmacological studies reporting that atropine injection delayed and/or decreased gastrointestinal tract motility, leading to constipation [9]. In mouse gastric and ileal tissues, the atropine-insensitive component of EFS-evoked neurogenic contraction was significantly increased in M_2_/M_3_ double-KO mice compared to wild-type mice [82,85,97], suggesting possible compensatory upregulation of non-cholinergic innervation, likely tachykinergic innervation.

However, the contributions of M_2_ and M_3_ receptors appear necessary in the colon for both propulsive motility and defecation. Kondo et al. [90] reported that atropine reduced 3 h faeces output in wild-type mice and that faeces evacuation during a 3 h period was clearly reduced in M_3_-KO and M_2_/M_3_ double-KO mice and more modestly reduced in M_2_-KO mice compared to wild-types. The time required to expel a 2 mm glass bead from the colon as an indication of propulsion force was also increased in KO mice (rank order of propulsion force: wild-type = M_2_-KO > M_3_-KO ≥ M_2_/M_3_ double-KO), suggesting that mAChRs, especially the M_3_ subtype, are necessary for colon propulsion and defecation in mice.

In a related study [42], the lack of TRPC4 and TRPC6 channels coupling mAChRs to depolarisation and contraction reduced the rate of charcoal transit within the small intestine in vivo, a result seemingly contradicting the null phenotype of M_3_-KO and M_2_/M_3_ double-KO mice (see above). This may be explained by compensatory TRPC4/TRPC6 channel stimulation by tachykinergic inputs to stimulate gastrointestinal motility.

Tanahashi et al. (2013) [111] found complex differences in the peristaltic movements among isolated small intestine from mAChR-KO and wild-type mice. Changes in intraluminal pressure (IP; representing circular muscle activity) and longitudinal muscle tension (LT) produced by luminal distension were simultaneously measured in the gut segments. The wild-type preparations responded to luminal distension with peristaltic movements characterised by synchronous rises in IP and LT occurring at a constant interval during the stimulus and sensitivity to the mAChR blocker atropine and the sodium channel blocker TTX, indicating the involvement of muscarinic transmission from enteric neurons. In contrast, only small and irregular fluctuations in IP and LT were produced by luminal distension in the majority of M_2_-KO preparations, and there was no organised peristalsis. This finding is consistent with the report of Schwörer and Klibinger (1988) [112] that pharmacological block of M_2_ receptors suppressed peristaltic activity in the guinea-pig small intestine. On the other hand, the peristaltic responses elicited in M_3_-KO preparations were characterised by repeated synchronous rises in IP and LT but with irregular periodicity. As in wild-type mice, these responses were sensitive to atropine and TTX. The M_2_/M_3_ double-KO preparations showed atropine-insensitive and TTX-sensitive peristaltic responses to luminal distension. Taken together, these results suggest that M_2_ and M_3_ receptors have distinct roles in peristaltic movement of the gut, with M_2_ receptors pivotal for generation of peristalsis and M_3_ receptors contributing to control of periodicity. It has been suggested that M_2_ and M_4_ receptors are located in the myenteric plexus of the mouse ileum and play an auto-inhibitory role in the release of acetylcholine [20,113]. It is well known that tackykinins are co-transmitters of excitatory motor neurons with ACh [114], implying existence of muscarinic auto-inhibition of the release of tackykinins as well as ACh. In the guinea-pig ileum, exogenous ACh stimulated peristalsis at low concentrations, but inhibited it at higher ones, suggesting the importance to maintain the appropriate concentrations of ACh and other neurotransmitters at the synaptic cleft [115]. Collectively, the autoinhibition of neurotransmitters by M_2_ may be essential to generate the peristalsis. Of note, the perturbation of peristaltic periodicity associated with M_3_ deficiency was mimicked by elimination of ICCs in the myenteric plexus (ICC-MYs) [111], suggesting that M_3_ receptors expressed by ICC-MYs are responsible for periodicity control. It has also been reported that ICC-MYs are involved in the propagation of peristaltic contraction rings along the mouse small intestine from the oral to the anal end [116]. Further study is needed to clarify where these M_2_ and M_3_ receptors are located since gut motility is regulated by activation of mAChRs in not only smooth muscle but also other cell types including enteric neurons and ICCs. However, there is a limitation of studies using global knockout of muscarinic receptor subtypes to address the issues because deficiency of muscarinic receptor subtypes in other cells may confound interpretation of the results. Mutant mice with mAChR subtype KO in specific cells should be useful to address these issues.

## 9. Concluding Remarks

The past decade has seen substantial progress in our understanding of M_2_ and M_3_ receptor functions in smooth muscle excitation and contraction due to the development of mAChR-KO and other genetically modified mice. The present article reviews many of those studies focusing on gastrointestinal smooth muscle. A major outcome of this research is the identification of three distinct pathways linking M_2_ and M_3_ receptor activation to TRPC cationic channel opening, the primary mechanism by which muscarinic agonists evoke depolarisation and contraction of gastrointestinal smooth muscle. The three signalling pathways identified are depicted in Figure 1. It is noteworthy that they all also converge on another effector of VDCCs to inhibit its activity and ensuing Ca^2+^ entry. Another major outcome is the elucidation of definitive roles for M_2_ and M_3_ receptors in inducing direct contraction or modulating contraction. A scheme depicting the predicted muscarinic mechanisms that elicit contraction is shown in Figure 2. It is of interest that both M_2_ and M_2_/M_3_ pathways activate Ca^2+^ entry only via VDCCs as a Ca^2+^ source for contraction, while the M_3_ pathway activates multiple mechanisms for mobilisation of intracellular Ca^2+^ and in addition increases the Ca^2+^ sensitivity of contractile proteins to enhance excitation–contraction coupling efficiency. Both M_2_ and M_2_/M_3_ pathways elicit contraction at low agonist concentrations, while the M_3_ pathway becomes predominant at higher agonist concentrations. Studies of mAChR-KO mice have also defined distinct roles for M_2_ and M_3_ receptors in heterologous desensitisation of contraction and gastrointestinal motility. Future progress can be expected through studies addressing whether the three muscarinic pathways (each organised with mAChRs, G-proteins and effectors) are compartmented separately, whether the M_2_/M_3_ pathway leads to significant InsP_3_/DAG formation and/or cyclic AMP inhibition and whether M_2_/M_3_ heterodimers actually exist. The roles of ICCs and neuronal mAChRs in regulating smooth muscle contraction also remain undefined. Extrapolating these results to human physiology and disease remains the ultimate goal. We believe these studies will facilitate the development of treatments for disorders of the gastrointestinal tract and other visceral smooth muscle organs.

## Figures and Tables

**Table 1:** E_max_ values for muscarinic agonist-induced contraction of visceral smooth muscle from various mAChR-mutant mice.

Tissue	Protocol/Agonist ^b^	E_max_ (% of Wild Type) ^a^				References
		M_2_-KO	M_3_-KO	M_2_/M_3_-KO	M_4_-KO	
Stomach fundus	Cumulative/CCh	96	44 *		95	Stengel et al. (2000; 2002) [80,81]
	Single dose/CCh	102	66 *	0 (relax.)		Kitazawa et al. (2007) [82]
	Cumulative/CCh	114	49 *	0 (relax.)		Kitazawa et al. (2007) [82]
Stomach antrum	Single dose/CCh	84	64 *	0 (relax.)		Kitazawa et al. (2007) [82]
	Cumulative/CCh	86	57 *	0 (relax.)		Kitazawa et al. (2007) [82]
Stomach body	Cumulative/CCh	95	42 *			Ruggieri and Braverman (2013) [83]
Ileum	Cumulative/CCh	75	25	0		Matsui et al. (2000; 2002) [84,85]
	Cumulative/Oxo.M	98				Matsui et al. (2003) [86]
	Single dose/CCh	103	62 *	0 (relax.)		Unno et al. (2005) [87]
	Single dose/ACh	102	43			Takeuchi et al. (2007) [88]
	Cumulative/Oxo.M	101	36 *			Griffin et al. (2009) [89]
Colon proximal	Single dose/CCh	66 *	34	0 (relax.)		Kondo et al. (2011) [90]
Colon distal	Single dose/CCh	65 *	21	0 (relax.)		Kondo et al. (2011) [90]
Trachea	Cumulative/CCh	86 *	44 *		82	Stengel et al. (2000; 2002) [80,81]
	Cumulative/Oxo.M	88				Matsui et al. (2003) [86]
Gallbladder	Cumulative/CCh	79	21		100	Stengel and Cohen (2002) [91]
Urinary bladder	Cumulative/CCh	95	5	0		Matsui et al. (2000; 2002) [84,85]
	Cumulative/Oxo.M	84				Matsui et al. (2003) [86]
	Cumulative/CCh	82	6 *		97	Stengel et al. (2000; 2002) [80,81]
	Cumulative/Oxo.M	89	15 *	0		Ehlert et al. (2005) [92]
	Single dose/CCh	77 *	7 *	0		#
Uterus	Cumulative/CCh	66 *	0	0		Kitazawa et al. (2008) [93]

^a^ Obtained directly from reported E_max_ values for mACh-mutant knockout (KO) strains and corresponding wild-type strain or estimated from concentration–response curves published for the KO and wild-type strains. ^b^ CCh: carbachol; ACh: acetylcholine; Oxo.M: oxotremorine-M. These agents were applied using a cumulative or single dose protocol. * Significant difference between mutant and wild-type mice. # Unpublished data (Komori, Tanahashi, Matsuyama, Unno).

**Table 2:** pEC_50_ values for muscarinic agonist-induced contraction of visceral smooth muscle from various mAChR-mutant mice.

Tissue	Protocol/Agonist	pEC_50_			References
		Wild Type: M_2_-KO	Wild Type: M_3_-KO	Wild Type: M_4_-KO	
Stomach fundus	Cumulative/CCh	6.68:6.39 *	6.54:6.71	6.76:6.70	Stengel et al. (2000; 2002) [80,81]
	Single dose/CCh	6.93:6.51 *	7.03:6.97		Kitazawa et al. (2007) [82]
	Cumulative/CCh	6.56:6.18 *	6.67:6.73		Kitazawa et al. (2007) [82]
Stomach antrum	Single dose/CCh	6.60:6.20 *	6.50:6.90		Kitazawa et al. (2007) [82]
	Cumulative/CCh	6.82:6.08 *	6.92:7.10		Kitazawa et al. (2007) [82]
Stomach body	Cumulative/CCh	6.1:5.7 *	6.1:6.5 *		Ruggieri and Braverman (2013) [83]
Ileum	Single dose/CCh	6.39:5.93 *	6.14:6.18		Unno et al. (2005) [87]
	Cumulative/Oxo.M	6.75:6.26 *	6.75:6.99 *		Griffin et al., (2009) [89]
	Cumulative/Oxo.M	6.70:6.38 *			Matsui et al. (2003) [86]
Colon proximal	Single dose/CCh	6.90:6.34 *			Kondo et al. (2011) [90]
Colon distal	Single dose/CCh	5.90:6.03			Kondo et al. (2011) [90]
Trachea	Cumulative/CCh	6.56:6.27 *	6.52:6.51	6.46:6.62	Stengel et al. (2000; 2002) [80,81]
	Cumulative/Oxo.M	6.94:6.86			Matsui et al. (2003) [86]
Urinary bladder	Cumulative/CCh	6.27:6.07 *	6.02:5.71 *	6.30:6.20	Stengel et al. (2000; 2002) [80,81]
	Cumulative/Oxo.M	6.54:6.31 *	6.54:6.60		Ehlert et al. (2005) [92]
	Cumulative/Oxo.M	6.58:6.41			Matsui et al. (2003) [86]
	Single dose/CCh	6.22:5.96	6.22:6.10		#

* Significant difference from the corresponding wild-type value. # Unpublished data (Komori, Tanahashi, Matsuyama, Unno). CCh: carbachol; Oxo.M: oxotremorine-M.

## Data Availability

Not applicable.

## Abbreviations

ACh	acetylcholine
BK channels	large conductance Ca^2+^-activated K^+^ channels
[Ca^2+^]_i_	intracellular Ca^2+^ concentration
cAMP	cyclic AMP
DAG	diacylglycerol
EC_50_	50% effective concentration
EFS	electrical field stimulation
EJP	excitatory junction potential
E_max_	maximum response
G-proteins	GTP binding proteins
I_ca_	Ca^2+^ current
ICCs	interstitial cells of Cajal
InsP_3_	inositol 1,4,5-trisphosphate
K_ATP_ channels	ATP-sensitive K^+^ channels
KO	knockout
*mIcat*	mAChR-mediated cationic current
muscarinic acetylcholine receptors	mAChRs
NO	nitric oxide
NPY	neuropeptide Y
pEC_50_	negative logarithm of EC_50_
PGF_2α_	prostaglandin F_2α_
PIP_2_	phosphatidylinositol 4,5-bisphosphate
PKC	protein kinase C
PLC	phospholipase C
PTX	pertussis toxin
RASSL	Receptor Solely by Synthetic Ligand
STOCs	spontaneous transient outward currents
TRPC channels	Transient receptor potential canonical channels
VDCCs	voltage-dependent Ca^2+^ channels
